# 4,7-Diphenyl-1,10-phenanthroline methanol hemisolvate

**DOI:** 10.1107/S1600536813014682

**Published:** 2013-06-08

**Authors:** João P. Martins, Pablo Martín-Ramos, Abílio J. F. N. Sobral, Manuela Ramos Silva

**Affiliations:** a CEMDRX, Physics Department, University of Coimbra, P-3004-516 Coimbra, Portugal; b Environmental Engineering Department, University of Valladolid, P-34004 Palencia, Spain; c Chemistry Department, University of Coimbra, P-3004 Coimbra, Portugal

## Abstract

The asymmetric unit of the title compound, C_24_H_16_N_2_·0.5CH_3_OH, is comprised of two independent bathophenanthroline mol­ecules (systematic name: 4,7-diphenyl-1,10-phenanthroline) and one methanol mol­ecule. The bathophenanthroline mol­ecules are not planar as there is a considerable rotation of all terminal phenyl rings with respect to the central phenanthroline units [dihedral angles in the range 52.21 (12)–62.14 (10)°]. In addition, a non-negligible torsion is apparent in one of the phenanthroline units: the angle between the mean planes of the two pyridine rings is 14.84 (13)°. The methanol solvent mol­ecule is linked to both N atoms of a bathophenanthroline mol­ecule through a bifurcated O—H⋯(N,N) hydrogen bond.

## Related literature

For background on aromatic *N*-donor lanthanide complexes, see: Martín-Ramos *et al.* (2013*a* ▶,*b* ▶); Reifernberger *et al.* (2005 ▶). For information on pure bathophenanthroline, see: Ceolin *et al.* (1979 ▶).
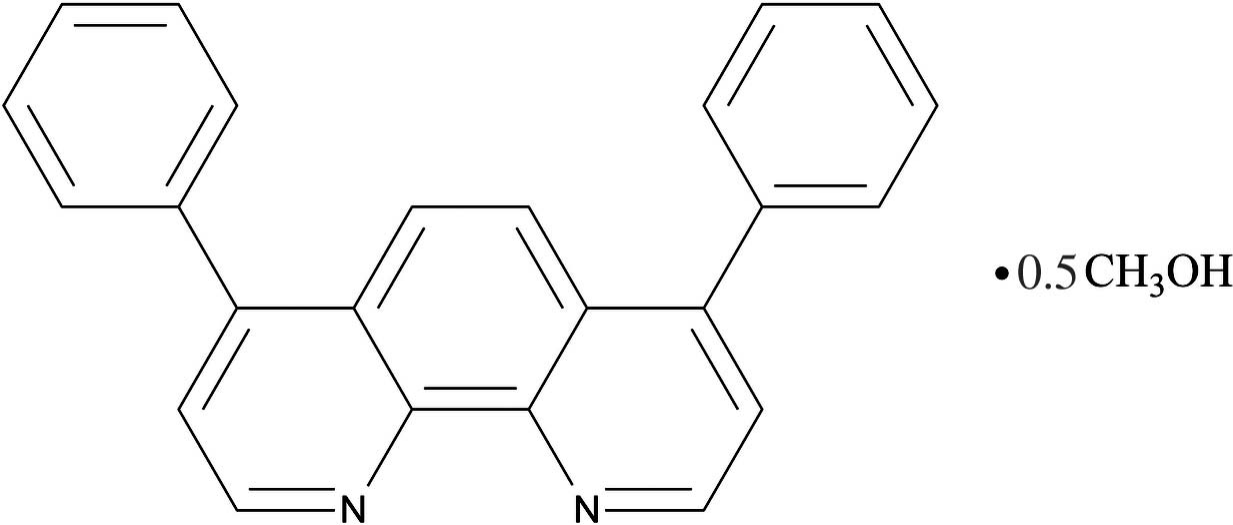


## Experimental

### 

#### Crystal data

C_24_H_16_N_2_·0.5CH_4_O
*M* *_r_* = 348.41
Triclinic, 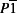

*a* = 7.2094 (3) Å
*b* = 14.8067 (6) Å
*c* = 18.1459 (7) Å
α = 109.797 (2)°
β = 99.921 (3)°
γ = 92.056 (3)°
*V* = 1785.99 (12) Å^3^
*Z* = 4
Mo *K*α radiation
μ = 0.08 mm^−1^
*T* = 293 K
0.50 × 0.22 × 0.10 mm

#### Data collection

Bruker APEX CCD area-detector diffractometer
Absorption correction: multi-scan (*SADABS*; Sheldrick, 2000 ▶) *T*_min_ = 0.865, *T*_max_ = 0.999
36139 measured reflections
6169 independent reflections
3407 reflections with *I* > 2σ(*I*)
*R*_int_ = 0.066

#### Refinement

*R*[*F* ^2^ > 2σ(*F* ^2^)] = 0.057
*wR*(*F* ^2^) = 0.170
*S* = 0.98
6160 reflections
489 parameters
H-atom parameters constrained
Δρ_max_ = 0.47 e Å^−3^
Δρ_min_ = −0.31 e Å^−3^

### 

Data collection: *SMART* (Bruker, 2003 ▶); cell refinement: *SAINT* (Bruker, 2003 ▶); data reduction: *SAINT*; program(s) used to solve structure: *SHELXS97* (Sheldrick, 2008 ▶); program(s) used to refine structure: *SHELXL97* (Sheldrick, 2008 ▶); molecular graphics: *ORTEPII* (Johnson, 1976 ▶); software used to prepare material for publication: *SHELXL97*.

## Supplementary Material

Crystal structure: contains datablock(s) global, I. DOI: 10.1107/S1600536813014682/fy2098sup1.cif

Structure factors: contains datablock(s) I. DOI: 10.1107/S1600536813014682/fy2098Isup2.hkl

Supplementary material file. DOI: 10.1107/S1600536813014682/fy2098Isup3.cml

Additional supplementary materials:  crystallographic information; 3D view; checkCIF report

## Figures and Tables

**Table 1:** Hydrogen-bond geometry (Å, °)

*D*—H⋯*A*	*D*—H	H⋯*A*	*D*⋯*A*	*D*—H⋯*A*
O1—H1*A*⋯N1^i^	0.82	2.13	2.873 (3)	151
O1—H1*A*⋯N2^i^	0.82	2.65	3.318 (3)	140

Symmetry code: (i) 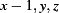.

**Fig. 1.**
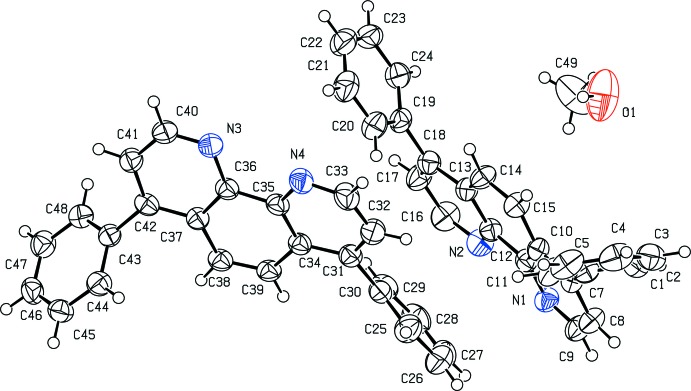
ORTEP plot of the title compound. Displacement ellipsoids are drawn at the 50% level.

**Fig. 2.**
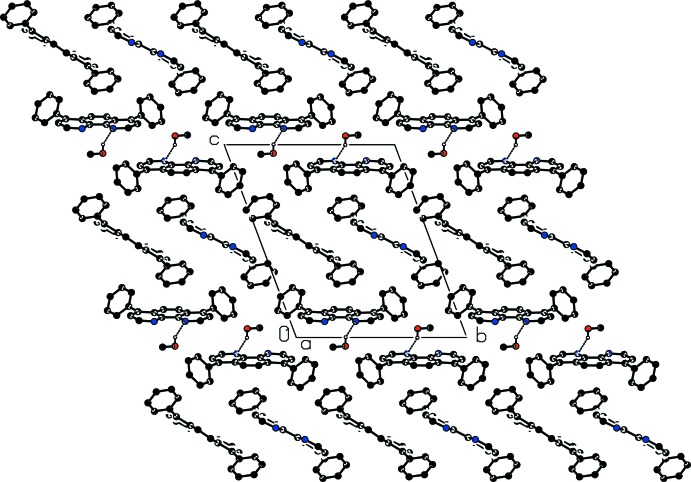
Packing of the molecules in the unit cell showing the H-bonds as dashed lines. H-atoms not involved in H-bonding were omitted for clarity.

## supplementary crystallographic information

### Comment

The title complex (I), Fig. 1, was obtained as part of scientific project to synthesize new Lanthanide coordination complexes and study their light emission properties in order to assess the suitability of such complexes as emissive layers in Organic Light Emitting Diodes (OLED) (Martín-Ramos *et al.*, 2013*a*, 2013*b*). The introduction of an aromatic N-donor in the lanthanide coordination sphere enlarges the absorption range of the excitation light and allows energy transfer to the lanthanide ion.

This complex was a result of an attempt to synthesize an Europium(*tris*(1,14- chlorophenyl-4,4,4- trifluoro-1,3-butanedionate)mono(bathophenanthroline)) coordination complex. Light pink crystals were recovered from this batch and after X-Ray diffraction structure determination the new bathophenanthroline solvate was revealed.

All bathophenanthroline phenyl groups presented in this compound show a considerable rotation with respect to the phenanthroline groups. This geometry can be explained by the single bond between the phenanthroline and phenyl rings. In bathophenanthroline molecule one the phenyl group C1—C5 shows a rotation of 58.04 (11)° with respect to the quasi-planar phenanthroline group C7—C18 whereas the other phenyl group C19—C24 shows a rotation of 52.21 (12)°. In the other bathophenanthroline molecule the angles between the mean plane of the non-planar phenanthroline group C31—C42 and the phenyl groups C25—C30 and C43—C48 are 56.49 (11)° and 62.14 (10)°, respectively. Furthermore, a non-negligible torsion is observed in one of the phenanthroline groups, the pseudo-torsion angle between C31..C32 and C41..C42 is 17.6 (5)° in the non-planar phenanthroline whereas in the quasi-planar one the angle between C7..C8 and C16..C17 is 4.6 (5)°. A similar torsion has been observed in the structure of the pure bathophenanthroline (Ceolin *et al.*, 1979)

The solvent methanol molecules are linked to one of the bathophenanthroline through O—H···N hidrogen bonds (Table 2).

When viewed along the *a* axis direction (Fig. 2) the packing diagram shows alternating layers. One layer contains the non-planar bathophenanthroline molecules packed so as to maximize π···π interaction between neighbouring molecules. The *Cg*1···*Cg*1^i^ distance is 4.2318 (17) Å (*Cg*1 is the centroid of the six-membered ring containing N3; ^i^: 1 - *x*, -*y*, 1 - *z*). The *Cg*2..*Cg*2^ii^ distance is 4.5147 (17) Å (*Cg*2 is the centroid of the six-membered ring containing N4; ^ii^: 1 - *x*, 1 - *y*, 1 - *z*). The other layer contains the quasi-planar bathophenanthroline molecules linked to the methanol molecules. The angle between the mean planes of the phenanthroline moieties of the quasi-planar and non-planar bathophenanthroline molecules is 32.59 (6)°.

### Experimental

First, 0.5 mmol of Europium(III) nitrate pentahydrate was dissolved in 20 ml of methanol, followed by the addition of 0.9 ml of potassium methoxide. This solution was left in reflux at 75°C for 10 minutes. Secondly, 1.5 mmol of 1,14 chlorophenyl-4,4,4-trifluoro-1,3-butanedionate was dissolved in 15 ml of methanol and added to the main solution. After decanting the resultant solution, 0.5 mmol of bathophenanthroline was dissolved in 10 ml of methanol and added to the main solution. The resultant solution was left in reflux at 75°C and 900 rpm for 4 h. After the evaporation process had been completed all the material from this batch was dissolved in 50% of methanol and 50% of chloroform with the intent of obtaining crystals. After the second evaporation process a yellow powder, which is believed to be the target complex was recovered alongside with some light pink crystals and some transparent crystals. The powder has proven to be amorphous while the light pink crystals turned out to be the title complex and the transparent ones potassium nitrate.

### Refinement

All hydrogen atoms bound to carbon atoms were placed at calculated positions and were treated as riding on the parent atoms with C—H = 0.93 Å and with *U*_iso_(H) = 1.2 *U*_eq_(C). The H atom belonging to the OH group was found in a difference electron density synthesis and subsequently refined with a fixed distance (0.82 Å) and angle (109.5°). In the final cycles of refinement, 14 bad outlier reflections, partially attenuated by the beamstop, were omitted.

### Crystal data

**Table:**

C_24_H_16_N_2_·0.5CH_4_O	*Z* = 4
*M**_r_* = 348.41	*F*(000) = 732
Triclinic, *P*1	*D*_x_ = 1.296 Mg m^−^^3^
Hall symbol: -P 1	Mo *K*α radiation, λ = 0.71073 Å
*a* = 7.2094 (3) Å	Cell parameters from 4091 reflections
*b* = 14.8067 (6) Å	θ = 3.1–23.9°
*c* = 18.1459 (7) Å	µ = 0.08 mm^−^^1^
α = 109.797 (2)°	*T* = 293 K
β = 99.921 (3)°	Prism, light pink
γ = 92.056 (3)°	0.50 × 0.22 × 0.10 mm
*V* = 1785.99 (12) Å^3^	

### Data collection

**Table:**

Bruker APEX CCD area-detector diffractometer	6169 independent reflections
Radiation source: fine-focus sealed tube	3407 reflections with *I* > 2σ(*I*)
Graphite monochromator	*R*_int_ = 0.066
φ and ω scans	θ_max_ = 24.9°, θ_min_ = 2.9°
Absorption correction: multi-scan (*SADABS*; Sheldrick, 2000)	*h* = −8→8
*T*_min_ = 0.865, *T*_max_ = 0.999	*k* = −17→17
36139 measured reflections	*l* = −21→21

### Refinement

**Table:**

Refinement on *F*^2^	Primary atom site location: structure-invariant direct methods
Least-squares matrix: full	Secondary atom site location: difference Fourier map
*R*[*F*^2^ > 2σ(*F*^2^)] = 0.057	Hydrogen site location: inferred from neighbouring sites
*wR*(*F*^2^) = 0.170	H-atom parameters constrained
*S* = 0.98	*w* = 1/[σ^2^(*F*_o_^2^) + (0.0921*P*)^2^] where *P* = (*F*_o_^2^ + 2*F*_c_^2^)/3
6160 reflections	(Δ/σ)_max_ < 0.001
489 parameters	Δρ_max_ = 0.47 e Å^−^^3^
0 restraints	Δρ_min_ = −0.31 e Å^−^^3^

### Special details

**Table:**

Geometry. All e.s.d.'s (except the e.s.d. in the dihedral angle between two l.s. planes) are estimated using the full covariance matrix. The cell e.s.d.'s are taken into account individually in the estimation of e.s.d.'s in distances, angles and torsion angles; correlations between e.s.d.'s in cell parameters are only used when they are defined by crystal symmetry. An approximate (isotropic) treatment of cell e.s.d.'s is used for estimating e.s.d.'s involving l.s. planes.
Refinement. Refinement of *F*^2^ against ALL reflections. The weighted *R*-factor *wR* and goodness of fit *S* are based on *F*^2^, conventional *R*-factors *R* are based on *F*, with *F* set to zero for negative *F*^2^. The threshold expression of *F*^2^ > σ(*F*^2^) is used only for calculating *R*-factors(gt) *etc*. and is not relevant to the choice of reflections for refinement. *R*-factors based on *F*^2^ are statistically about twice as large as those based on *F*, and *R*- factors based on ALL data will be even larger.

### Fractional atomic coordinates and isotropic or equivalent isotropic displacement parameters (Å^2^)

**Table:**

	*x*	*y*	*z*	*U*_iso_*/*U*_eq_	
N1	0.9651 (3)	0.38369 (17)	0.08387 (13)	0.0448 (6)	
N2	0.9368 (3)	0.20160 (17)	0.08314 (14)	0.0487 (6)	
N3	0.4206 (3)	0.17336 (17)	0.52893 (14)	0.0458 (6)	
N4	0.4034 (3)	0.31882 (17)	0.46951 (14)	0.0478 (6)	
C11	0.8091 (3)	0.35319 (19)	0.10489 (15)	0.0378 (7)	
C13	0.6376 (4)	0.22144 (18)	0.12922 (15)	0.0382 (7)	
C12	0.7945 (3)	0.25552 (19)	0.10455 (15)	0.0381 (7)	
C35	0.5712 (3)	0.28105 (19)	0.47868 (15)	0.0396 (7)	
C15	0.5014 (4)	0.37245 (19)	0.14451 (16)	0.0437 (7)	
H15	0.4008	0.4093	0.1548	0.052*	
C37	0.7644 (3)	0.18523 (18)	0.54400 (15)	0.0371 (6)	
C10	0.6667 (3)	0.41336 (18)	0.12773 (15)	0.0373 (6)	
C38	0.9256 (3)	0.22017 (19)	0.52145 (16)	0.0407 (7)	
H38	1.0448	0.2041	0.5387	0.049*	
C43	0.9538 (3)	0.10005 (19)	0.62919 (15)	0.0380 (7)	
C8	0.8483 (4)	0.5384 (2)	0.10858 (17)	0.0494 (8)	
H8	0.8677	0.6010	0.1090	0.059*	
C42	0.7740 (3)	0.12261 (19)	0.58904 (15)	0.0391 (7)	
C14	0.4895 (4)	0.28238 (19)	0.14559 (16)	0.0434 (7)	
H14	0.3813	0.2588	0.1574	0.052*	
C41	0.6061 (4)	0.0850 (2)	0.59819 (16)	0.0440 (7)	
H41	0.6059	0.0423	0.6258	0.053*	
C36	0.5849 (4)	0.2110 (2)	0.51828 (15)	0.0395 (7)	
C30	0.8575 (4)	0.3986 (2)	0.37038 (16)	0.0446 (7)	
C46	1.2725 (4)	0.0591 (2)	0.71830 (18)	0.0515 (8)	
H46	1.3776	0.0453	0.7487	0.062*	
C34	0.7317 (3)	0.30917 (19)	0.45299 (15)	0.0386 (7)	
C31	0.7088 (4)	0.3727 (2)	0.40934 (16)	0.0420 (7)	
C19	0.4808 (4)	0.0849 (2)	0.16078 (17)	0.0422 (7)	
C7	0.6889 (4)	0.50933 (19)	0.12980 (16)	0.0395 (7)	
C6	0.5452 (4)	0.57764 (18)	0.15246 (17)	0.0416 (7)	
C39	0.9087 (4)	0.27546 (19)	0.47600 (16)	0.0407 (7)	
H39	1.0148	0.2925	0.4589	0.049*	
C33	0.3949 (4)	0.3835 (2)	0.43481 (18)	0.0522 (8)	
H33	0.2846	0.4139	0.4320	0.063*	
C17	0.7826 (4)	0.0759 (2)	0.11293 (18)	0.0525 (8)	
H17	0.7858	0.0143	0.1156	0.063*	
C18	0.6352 (4)	0.12801 (19)	0.13395 (16)	0.0423 (7)	
C40	0.4355 (4)	0.1113 (2)	0.56576 (17)	0.0467 (7)	
H40	0.3241	0.0824	0.5708	0.056*	
C24	0.3922 (4)	−0.0055 (2)	0.11419 (18)	0.0505 (8)	
H24	0.4247	−0.0376	0.0652	0.061*	
C32	0.5389 (4)	0.4100 (2)	0.40190 (18)	0.0511 (8)	
H32	0.5196	0.4532	0.3748	0.061*	
C9	0.9801 (4)	0.4735 (2)	0.08660 (17)	0.0511 (8)	
H9	1.0871	0.4952	0.0727	0.061*	
C5	0.4975 (4)	0.6028 (2)	0.22745 (19)	0.0543 (8)	
H5	0.5533	0.5757	0.2641	0.065*	
C44	1.0808 (4)	0.1737 (2)	0.68614 (17)	0.0460 (7)	
H44	1.0597	0.2377	0.6940	0.055*	
C47	1.1523 (4)	−0.0142 (2)	0.66091 (18)	0.0514 (8)	
H47	1.1783	−0.0778	0.6513	0.062*	
C45	1.2371 (4)	0.1536 (2)	0.73091 (17)	0.0489 (7)	
H45	1.3190	0.2037	0.7697	0.059*	
C1	0.4597 (4)	0.61961 (19)	0.10000 (18)	0.0484 (7)	
H1	0.4914	0.6041	0.0499	0.058*	
C48	0.9925 (4)	0.0054 (2)	0.61697 (17)	0.0452 (7)	
H48	0.9103	−0.0452	0.5789	0.054*	
C29	0.9413 (4)	0.3288 (2)	0.31805 (17)	0.0529 (8)	
H29	0.9138	0.2640	0.3104	0.063*	
C25	0.9042 (4)	0.4949 (2)	0.38128 (18)	0.0582 (8)	
H25	0.8494	0.5428	0.4163	0.070*	
C16	0.9279 (4)	0.1146 (2)	0.08762 (18)	0.0553 (8)	
H16	1.0248	0.0767	0.0730	0.066*	
C26	1.0302 (5)	0.5202 (3)	0.3410 (2)	0.0672 (9)	
H26	1.0613	0.5850	0.3496	0.081*	
C2	0.3278 (4)	0.6843 (2)	0.1204 (2)	0.0593 (9)	
H2	0.2703	0.7111	0.0838	0.071*	
C23	0.2552 (4)	−0.0490 (2)	0.1397 (2)	0.0569 (8)	
H23	0.1957	−0.1098	0.1076	0.068*	
C27	1.1099 (5)	0.4507 (3)	0.2884 (2)	0.0679 (10)	
H27	1.1932	0.4679	0.2605	0.082*	
C28	1.0662 (4)	0.3554 (3)	0.27703 (18)	0.0616 (9)	
H28	1.1209	0.3081	0.2414	0.074*	
C22	0.2072 (4)	−0.0025 (2)	0.2121 (2)	0.0620 (9)	
H22	0.1156	−0.0319	0.2293	0.074*	
C20	0.4307 (4)	0.1310 (2)	0.23362 (19)	0.0542 (8)	
H20	0.4890	0.1921	0.2658	0.065*	
C21	0.2940 (5)	0.0869 (3)	0.2590 (2)	0.0615 (9)	
H21	0.2615	0.1183	0.3081	0.074*	
C4	0.3653 (5)	0.6691 (2)	0.2472 (2)	0.0648 (10)	
H4	0.3338	0.6863	0.2974	0.078*	
C3	0.2817 (4)	0.7090 (2)	0.1939 (2)	0.0644 (10)	
H3	0.1934	0.7529	0.2077	0.077*	
O1	0.1335 (5)	0.2689 (2)	−0.04489 (19)	0.1219 (11)	
H1A	0.0924	0.2841	−0.0034	0.183*	
C49	0.2363 (5)	0.1943 (3)	−0.0499 (2)	0.0913 (13)	
H49A	0.3675	0.2177	−0.0295	0.137*	
H49B	0.2200	0.1528	−0.1048	0.137*	
H49C	0.1950	0.1588	−0.0191	0.137*	

### Atomic displacement parameters (Å^2^)

**Table:**

	*U*^11^	*U*^22^	*U*^33^	*U*^12^	*U*^13^	*U*^23^
N1	0.0382 (13)	0.0512 (16)	0.0519 (15)	0.0066 (11)	0.0152 (11)	0.0239 (12)
N2	0.0446 (14)	0.0482 (15)	0.0581 (16)	0.0158 (12)	0.0198 (12)	0.0186 (13)
N3	0.0329 (13)	0.0593 (16)	0.0508 (15)	0.0039 (11)	0.0114 (11)	0.0250 (13)
N4	0.0353 (13)	0.0610 (16)	0.0539 (16)	0.0102 (12)	0.0083 (11)	0.0287 (14)
C11	0.0356 (15)	0.0444 (17)	0.0347 (15)	0.0043 (13)	0.0089 (12)	0.0147 (13)
C13	0.0393 (15)	0.0373 (16)	0.0393 (16)	0.0062 (13)	0.0079 (12)	0.0146 (13)
C12	0.0350 (15)	0.0417 (17)	0.0388 (16)	0.0067 (13)	0.0087 (12)	0.0147 (13)
C35	0.0291 (14)	0.0498 (17)	0.0396 (16)	0.0026 (13)	0.0052 (12)	0.0162 (14)
C15	0.0401 (16)	0.0398 (17)	0.0555 (19)	0.0122 (13)	0.0184 (14)	0.0172 (14)
C37	0.0331 (15)	0.0419 (16)	0.0368 (15)	0.0059 (12)	0.0100 (12)	0.0129 (13)
C10	0.0396 (15)	0.0371 (16)	0.0371 (16)	0.0047 (13)	0.0099 (12)	0.0140 (13)
C38	0.0290 (14)	0.0496 (17)	0.0431 (17)	0.0046 (12)	0.0086 (12)	0.0148 (15)
C43	0.0341 (15)	0.0453 (17)	0.0381 (16)	0.0049 (13)	0.0122 (13)	0.0164 (14)
C8	0.0496 (18)	0.0449 (18)	0.062 (2)	0.0049 (14)	0.0195 (15)	0.0247 (16)
C42	0.0321 (15)	0.0458 (16)	0.0360 (16)	0.0070 (13)	0.0077 (12)	0.0093 (14)
C14	0.0388 (15)	0.0417 (17)	0.0552 (19)	0.0048 (13)	0.0201 (14)	0.0188 (15)
C41	0.0387 (16)	0.0523 (18)	0.0456 (18)	0.0047 (14)	0.0113 (13)	0.0215 (15)
C36	0.0314 (15)	0.0503 (17)	0.0358 (16)	0.0013 (13)	0.0077 (12)	0.0138 (14)
C30	0.0396 (16)	0.055 (2)	0.0392 (17)	−0.0008 (14)	0.0038 (13)	0.0194 (15)
C46	0.0383 (16)	0.073 (2)	0.053 (2)	0.0088 (16)	0.0061 (15)	0.0356 (18)
C34	0.0332 (15)	0.0455 (16)	0.0353 (15)	0.0027 (13)	0.0066 (12)	0.0121 (14)
C31	0.0400 (16)	0.0469 (17)	0.0378 (16)	0.0001 (13)	0.0064 (13)	0.0143 (14)
C19	0.0426 (16)	0.0388 (17)	0.0517 (19)	0.0087 (14)	0.0096 (14)	0.0233 (15)
C7	0.0411 (16)	0.0402 (16)	0.0395 (16)	0.0044 (13)	0.0092 (13)	0.0165 (13)
C6	0.0433 (16)	0.0332 (15)	0.0491 (18)	0.0009 (13)	0.0116 (14)	0.0146 (14)
C39	0.0308 (15)	0.0492 (17)	0.0418 (16)	0.0008 (13)	0.0102 (12)	0.0146 (15)
C33	0.0414 (17)	0.062 (2)	0.058 (2)	0.0147 (15)	0.0077 (15)	0.0271 (17)
C17	0.0537 (19)	0.0428 (18)	0.070 (2)	0.0109 (15)	0.0160 (16)	0.0282 (16)
C18	0.0417 (16)	0.0409 (16)	0.0444 (17)	0.0057 (13)	0.0059 (13)	0.0160 (14)
C40	0.0348 (16)	0.0578 (19)	0.0504 (18)	0.0006 (14)	0.0114 (14)	0.0217 (16)
C24	0.0484 (18)	0.0505 (19)	0.0548 (19)	0.0073 (15)	0.0075 (15)	0.0223 (16)
C32	0.0429 (17)	0.059 (2)	0.0576 (19)	0.0071 (15)	0.0100 (15)	0.0287 (16)
C9	0.0453 (17)	0.055 (2)	0.064 (2)	0.0058 (15)	0.0194 (15)	0.0293 (17)
C5	0.068 (2)	0.0423 (17)	0.061 (2)	0.0074 (15)	0.0273 (17)	0.0214 (16)
C44	0.0414 (16)	0.0473 (18)	0.0503 (19)	0.0071 (14)	0.0093 (14)	0.0179 (15)
C47	0.0499 (18)	0.0523 (19)	0.059 (2)	0.0123 (16)	0.0136 (16)	0.0260 (17)
C45	0.0407 (17)	0.058 (2)	0.0448 (18)	−0.0028 (15)	0.0047 (14)	0.0167 (15)
C1	0.0516 (18)	0.0394 (17)	0.0503 (18)	0.0068 (14)	0.0085 (15)	0.0113 (15)
C48	0.0429 (17)	0.0459 (18)	0.0463 (18)	0.0021 (14)	0.0082 (14)	0.0161 (15)
C29	0.0464 (17)	0.064 (2)	0.0428 (18)	0.0016 (15)	0.0075 (15)	0.0132 (16)
C25	0.060 (2)	0.062 (2)	0.057 (2)	0.0040 (17)	0.0197 (17)	0.0235 (17)
C16	0.0501 (18)	0.050 (2)	0.072 (2)	0.0204 (15)	0.0214 (16)	0.0229 (17)
C26	0.070 (2)	0.073 (2)	0.068 (2)	−0.0039 (19)	0.0191 (19)	0.034 (2)
C2	0.0540 (19)	0.0483 (19)	0.070 (2)	0.0077 (16)	0.0016 (17)	0.0188 (18)
C23	0.0486 (18)	0.0482 (19)	0.076 (2)	−0.0022 (15)	−0.0001 (17)	0.0304 (18)
C27	0.057 (2)	0.107 (3)	0.051 (2)	−0.006 (2)	0.0099 (17)	0.043 (2)
C28	0.0512 (19)	0.089 (3)	0.0406 (19)	0.0080 (18)	0.0126 (15)	0.0160 (18)
C22	0.0494 (19)	0.069 (2)	0.081 (3)	0.0040 (18)	0.0148 (18)	0.043 (2)
C20	0.063 (2)	0.0447 (18)	0.060 (2)	0.0063 (15)	0.0154 (17)	0.0231 (16)
C21	0.064 (2)	0.071 (2)	0.062 (2)	0.0113 (18)	0.0231 (18)	0.034 (2)
C4	0.078 (2)	0.0450 (19)	0.074 (2)	0.0027 (18)	0.040 (2)	0.0113 (18)
C3	0.053 (2)	0.045 (2)	0.096 (3)	0.0123 (16)	0.026 (2)	0.019 (2)
O1	0.165 (3)	0.133 (3)	0.115 (3)	0.070 (2)	0.075 (2)	0.074 (2)
C49	0.092 (3)	0.078 (3)	0.074 (3)	0.049 (2)	0.004 (2)	−0.010 (2)

### Geometric parameters (Å, º)

**Table:**

N1—C9	1.314 (3)	C39—H39	0.9300
N1—C11	1.358 (3)	C33—C32	1.390 (4)
N2—C16	1.318 (3)	C33—H33	0.9300
N2—C12	1.352 (3)	C17—C18	1.367 (3)
N3—C40	1.304 (3)	C17—C16	1.392 (4)
N3—C36	1.362 (3)	C17—H17	0.9300
N4—C33	1.311 (3)	C40—H40	0.9300
N4—C35	1.359 (3)	C24—C23	1.384 (4)
C11—C10	1.407 (3)	C24—H24	0.9300
C11—C12	1.444 (4)	C32—H32	0.9300
C13—C18	1.414 (3)	C9—H9	0.9300
C13—C12	1.421 (3)	C5—C4	1.396 (4)
C13—C14	1.428 (3)	C5—H5	0.9300
C35—C34	1.418 (3)	C44—C45	1.375 (4)
C35—C36	1.446 (4)	C44—H44	0.9300
C15—C14	1.340 (3)	C47—C48	1.383 (4)
C15—C10	1.442 (3)	C47—H47	0.9300
C15—H15	0.9300	C45—H45	0.9300
C37—C36	1.410 (3)	C1—C2	1.380 (4)
C37—C42	1.425 (4)	C1—H1	0.9300
C37—C38	1.432 (3)	C48—H48	0.9300
C10—C7	1.411 (3)	C29—C28	1.387 (4)
C38—C39	1.340 (4)	C29—H29	0.9300
C38—H38	0.9300	C25—C26	1.374 (4)
C43—C48	1.389 (4)	C25—H25	0.9300
C43—C44	1.391 (4)	C16—H16	0.9300
C43—C42	1.481 (3)	C26—C27	1.367 (4)
C8—C7	1.373 (3)	C26—H26	0.9300
C8—C9	1.386 (4)	C2—C3	1.362 (4)
C8—H8	0.9300	C2—H2	0.9300
C42—C41	1.374 (3)	C23—C22	1.372 (4)
C14—H14	0.9300	C23—H23	0.9300
C41—C40	1.396 (4)	C27—C28	1.372 (4)
C41—H41	0.9300	C27—H27	0.9300
C30—C29	1.383 (4)	C28—H28	0.9300
C30—C25	1.391 (4)	C22—C21	1.364 (4)
C30—C31	1.487 (4)	C22—H22	0.9300
C46—C47	1.367 (4)	C20—C21	1.387 (4)
C46—C45	1.381 (4)	C20—H20	0.9300
C46—H46	0.9300	C21—H21	0.9300
C34—C31	1.418 (4)	C4—C3	1.364 (4)
C34—C39	1.434 (3)	C4—H4	0.9300
C31—C32	1.365 (4)	C3—H3	0.9300
C19—C24	1.379 (4)	O1—C49	1.339 (4)
C19—C20	1.384 (4)	O1—H1A	0.8200
C19—C18	1.493 (4)	C49—H49A	0.9600
C7—C6	1.485 (3)	C49—H49B	0.9600
C6—C1	1.378 (4)	C49—H49C	0.9600
C6—C5	1.391 (4)		
			
C9—N1—C11	117.0 (2)	C13—C18—C19	123.1 (2)
C16—N2—C12	117.1 (2)	N3—C40—C41	125.0 (3)
C40—N3—C36	116.6 (2)	N3—C40—H40	117.5
C33—N4—C35	116.6 (2)	C41—C40—H40	117.5
N1—C11—C10	122.5 (2)	C19—C24—C23	120.6 (3)
N1—C11—C12	117.3 (2)	C19—C24—H24	119.7
C10—C11—C12	120.2 (2)	C23—C24—H24	119.7
C18—C13—C12	118.0 (2)	C31—C32—C33	120.0 (3)
C18—C13—C14	123.9 (2)	C31—C32—H32	120.0
C12—C13—C14	118.1 (2)	C33—C32—H32	120.0
N2—C12—C13	123.0 (2)	N1—C9—C8	124.9 (3)
N2—C12—C11	117.5 (2)	N1—C9—H9	117.6
C13—C12—C11	119.5 (2)	C8—C9—H9	117.6
N4—C35—C34	122.9 (3)	C6—C5—C4	119.4 (3)
N4—C35—C36	117.5 (2)	C6—C5—H5	120.3
C34—C35—C36	119.6 (2)	C4—C5—H5	120.3
C14—C15—C10	121.4 (2)	C45—C44—C43	121.1 (3)
C14—C15—H15	119.3	C45—C44—H44	119.5
C10—C15—H15	119.3	C43—C44—H44	119.5
C36—C37—C42	117.7 (2)	C46—C47—C48	120.6 (3)
C36—C37—C38	118.8 (2)	C46—C47—H47	119.7
C42—C37—C38	123.5 (2)	C48—C47—H47	119.7
C11—C10—C7	118.5 (2)	C44—C45—C46	120.0 (3)
C11—C10—C15	118.2 (2)	C44—C45—H45	120.0
C7—C10—C15	123.2 (2)	C46—C45—H45	120.0
C39—C38—C37	121.5 (2)	C6—C1—C2	121.2 (3)
C39—C38—H38	119.3	C6—C1—H1	119.4
C37—C38—H38	119.3	C2—C1—H1	119.4
C48—C43—C44	118.1 (3)	C47—C48—C43	120.5 (3)
C48—C43—C42	121.4 (2)	C47—C48—H48	119.7
C44—C43—C42	120.2 (2)	C43—C48—H48	119.7
C7—C8—C9	119.3 (3)	C30—C29—C28	120.0 (3)
C7—C8—H8	120.3	C30—C29—H29	120.0
C9—C8—H8	120.3	C28—C29—H29	120.0
C41—C42—C37	117.5 (2)	C26—C25—C30	121.0 (3)
C41—C42—C43	118.8 (3)	C26—C25—H25	119.5
C37—C42—C43	123.6 (2)	C30—C25—H25	119.5
C15—C14—C13	122.1 (2)	N2—C16—C17	124.1 (3)
C15—C14—H14	118.9	N2—C16—H16	117.9
C13—C14—H14	118.9	C17—C16—H16	117.9
C42—C41—C40	119.6 (3)	C27—C26—C25	120.3 (3)
C42—C41—H41	120.2	C27—C26—H26	119.9
C40—C41—H41	120.2	C25—C26—H26	119.9
N3—C36—C37	123.3 (3)	C3—C2—C1	120.2 (3)
N3—C36—C35	117.4 (2)	C3—C2—H2	119.9
C37—C36—C35	119.3 (2)	C1—C2—H2	119.9
C29—C30—C25	118.4 (3)	C22—C23—C24	120.1 (3)
C29—C30—C31	121.6 (3)	C22—C23—H23	119.9
C25—C30—C31	119.8 (3)	C24—C23—H23	119.9
C47—C46—C45	119.8 (3)	C26—C27—C28	119.6 (3)
C47—C46—H46	120.1	C26—C27—H27	120.2
C45—C46—H46	120.1	C28—C27—H27	120.2
C35—C34—C31	117.8 (2)	C27—C28—C29	120.7 (3)
C35—C34—C39	118.1 (3)	C27—C28—H28	119.6
C31—C34—C39	124.0 (2)	C29—C28—H28	119.6
C32—C31—C34	117.5 (2)	C21—C22—C23	119.9 (3)
C32—C31—C30	119.0 (3)	C21—C22—H22	120.0
C34—C31—C30	123.5 (2)	C23—C22—H22	120.0
C24—C19—C20	118.6 (3)	C19—C20—C21	120.4 (3)
C24—C19—C18	119.6 (3)	C19—C20—H20	119.8
C20—C19—C18	121.7 (3)	C21—C20—H20	119.8
C8—C7—C10	117.8 (2)	C22—C21—C20	120.2 (3)
C8—C7—C6	119.9 (2)	C22—C21—H21	119.9
C10—C7—C6	122.3 (2)	C20—C21—H21	119.9
C1—C6—C5	118.5 (3)	C3—C4—C5	120.9 (3)
C1—C6—C7	120.4 (2)	C3—C4—H4	119.6
C5—C6—C7	121.1 (3)	C5—C4—H4	119.6
C38—C39—C34	121.8 (2)	C4—C3—C2	119.8 (3)
C38—C39—H39	119.1	C4—C3—H3	120.1
C34—C39—H39	119.1	C2—C3—H3	120.1
N4—C33—C32	124.7 (3)	C49—O1—H1A	109.5
N4—C33—H33	117.6	O1—C49—H49A	109.5
C32—C33—H33	117.6	O1—C49—H49B	109.5
C18—C17—C16	120.3 (3)	H49A—C49—H49B	109.5
C18—C17—H17	119.8	O1—C49—H49C	109.5
C16—C17—H17	119.8	H49A—C49—H49C	109.5
C17—C18—C13	117.6 (2)	H49B—C49—H49C	109.5
C17—C18—C19	119.3 (2)		
			
C9—N1—C11—C10	0.8 (4)	C11—C10—C7—C6	179.4 (2)
C9—N1—C11—C12	−178.0 (2)	C15—C10—C7—C6	2.2 (4)
C16—N2—C12—C13	−1.5 (4)	C8—C7—C6—C1	57.8 (4)
C16—N2—C12—C11	176.0 (3)	C10—C7—C6—C1	−121.1 (3)
C18—C13—C12—N2	2.2 (4)	C8—C7—C6—C5	−120.5 (3)
C14—C13—C12—N2	−176.1 (2)	C10—C7—C6—C5	60.7 (4)
C18—C13—C12—C11	−175.3 (2)	C37—C38—C39—C34	5.2 (4)
C14—C13—C12—C11	6.4 (4)	C35—C34—C39—C38	−1.0 (4)
N1—C11—C12—N2	−0.2 (4)	C31—C34—C39—C38	175.8 (3)
C10—C11—C12—N2	−179.1 (2)	C35—N4—C33—C32	5.1 (4)
N1—C11—C12—C13	177.5 (2)	C16—C17—C18—C13	−0.3 (4)
C10—C11—C12—C13	−1.5 (4)	C16—C17—C18—C19	179.9 (3)
C33—N4—C35—C34	0.3 (4)	C12—C13—C18—C17	−1.2 (4)
C33—N4—C35—C36	178.8 (2)	C14—C13—C18—C17	177.0 (3)
N1—C11—C10—C7	−0.9 (4)	C12—C13—C18—C19	178.6 (2)
C12—C11—C10—C7	178.0 (2)	C14—C13—C18—C19	−3.2 (4)
N1—C11—C10—C15	176.5 (2)	C24—C19—C18—C17	−51.8 (4)
C12—C11—C10—C15	−4.7 (4)	C20—C19—C18—C17	124.6 (3)
C14—C15—C10—C11	6.0 (4)	C24—C19—C18—C13	128.4 (3)
C14—C15—C10—C7	−176.8 (3)	C20—C19—C18—C13	−55.2 (4)
C36—C37—C38—C39	−0.8 (4)	C36—N3—C40—C41	3.5 (4)
C42—C37—C38—C39	176.9 (2)	C42—C41—C40—N3	−2.9 (4)
C36—C37—C42—C41	5.6 (4)	C20—C19—C24—C23	0.3 (4)
C38—C37—C42—C41	−172.2 (2)	C18—C19—C24—C23	176.8 (2)
C36—C37—C42—C43	−171.4 (2)	C34—C31—C32—C33	−1.5 (4)
C38—C37—C42—C43	10.8 (4)	C30—C31—C32—C33	177.2 (3)
C48—C43—C42—C41	55.3 (3)	N4—C33—C32—C31	−4.6 (5)
C44—C43—C42—C41	−118.6 (3)	C11—N1—C9—C8	−0.6 (4)
C48—C43—C42—C37	−127.7 (3)	C7—C8—C9—N1	0.3 (5)
C44—C43—C42—C37	58.4 (3)	C1—C6—C5—C4	0.1 (4)
C10—C15—C14—C13	−0.9 (4)	C7—C6—C5—C4	178.4 (3)
C18—C13—C14—C15	176.5 (3)	C48—C43—C44—C45	−2.0 (4)
C12—C13—C14—C15	−5.4 (4)	C42—C43—C44—C45	172.1 (2)
C37—C42—C41—C40	−1.9 (4)	C45—C46—C47—C48	−2.0 (4)
C43—C42—C41—C40	175.2 (2)	C43—C44—C45—C46	1.6 (4)
C40—N3—C36—C37	0.8 (4)	C47—C46—C45—C44	0.4 (4)
C40—N3—C36—C35	−179.0 (2)	C5—C6—C1—C2	−0.8 (4)
C42—C37—C36—N3	−5.3 (4)	C7—C6—C1—C2	−179.1 (2)
C38—C37—C36—N3	172.6 (2)	C46—C47—C48—C43	1.5 (4)
C42—C37—C36—C35	174.5 (2)	C44—C43—C48—C47	0.5 (4)
C38—C37—C36—C35	−7.6 (4)	C42—C43—C48—C47	−173.6 (2)
N4—C35—C36—N3	13.0 (4)	C25—C30—C29—C28	−1.0 (4)
C34—C35—C36—N3	−168.5 (2)	C31—C30—C29—C28	173.9 (3)
N4—C35—C36—C37	−166.8 (2)	C29—C30—C25—C26	0.1 (4)
C34—C35—C36—C37	11.7 (4)	C31—C30—C25—C26	−174.8 (3)
N4—C35—C34—C31	−6.0 (4)	C12—N2—C16—C17	−0.1 (4)
C36—C35—C34—C31	175.6 (2)	C18—C17—C16—N2	1.0 (5)
N4—C35—C34—C39	171.0 (2)	C30—C25—C26—C27	1.0 (5)
C36—C35—C34—C39	−7.4 (4)	C6—C1—C2—C3	1.0 (4)
C35—C34—C31—C32	6.4 (4)	C19—C24—C23—C22	−0.5 (4)
C39—C34—C31—C32	−170.5 (2)	C25—C26—C27—C28	−1.2 (5)
C35—C34—C31—C30	−172.3 (2)	C26—C27—C28—C29	0.3 (5)
C39—C34—C31—C30	10.9 (4)	C30—C29—C28—C27	0.8 (4)
C29—C30—C31—C32	−125.5 (3)	C24—C23—C22—C21	0.3 (5)
C25—C30—C31—C32	49.3 (4)	C24—C19—C20—C21	0.0 (4)
C29—C30—C31—C34	53.2 (4)	C18—C19—C20—C21	−176.4 (3)
C25—C30—C31—C34	−132.0 (3)	C23—C22—C21—C20	0.1 (5)
C9—C8—C7—C10	−0.3 (4)	C19—C20—C21—C22	−0.2 (4)
C9—C8—C7—C6	−179.2 (3)	C6—C5—C4—C3	0.4 (5)
C11—C10—C7—C8	0.6 (4)	C5—C4—C3—C2	−0.2 (5)
C15—C10—C7—C8	−176.6 (3)	C1—C2—C3—C4	−0.4 (5)

### Hydrogen-bond geometry (Å, º)

**Table:**

*D*—H···*A*	*D*—H	H···*A*	*D*···*A*	*D*—H···*A*
O1—H1*A*···N1^i^	0.82	2.13	2.873 (3)	151
O1—H1*A*···N2^i^	0.82	2.65	3.318 (3)	140

Symmetry code: (i) *x*−1, *y*, *z*.
